# Supplementary material for: Dendritic normalisation improves learning in sparsely connected artificial neural networks
Source: PLoS Comput Biol. 2021 Aug 9;17(8):e1009202. doi: 10.1371/journal.pcbi.1009202 (PMC8407571; doi:10.1371/journal.pcbi.1009202)
Supplement: S1 Fig — A supplementary figure showing the performance of dendritic normalisation at different levels of sparsity. (PDF) [file pcbi.1009202.s001.pdf]

Supplementary Figure: Dendritic normalisation improves learning in sparsely connected artificial neural networks

A D Bird[1,2,3*], P Jedlicka[2,3], H Cuntz[1,2]

[1] Frankfurt Institute for Advanced Studies, Frankfurt-am-Main, Germany
[2] Ernst Strüngmann Institute (ESI) for Neuroscience in cooperation with the Max Planck Society, Frankfurt-am-Main, Germany
[3] ICAR3R-Interdisciplinary Centre for 3Rs in Animal Research, Faculty of Medicine, Justus Liebig University Giessen, Giessen, Germany

*bird@fias.uni-frankfurt.de

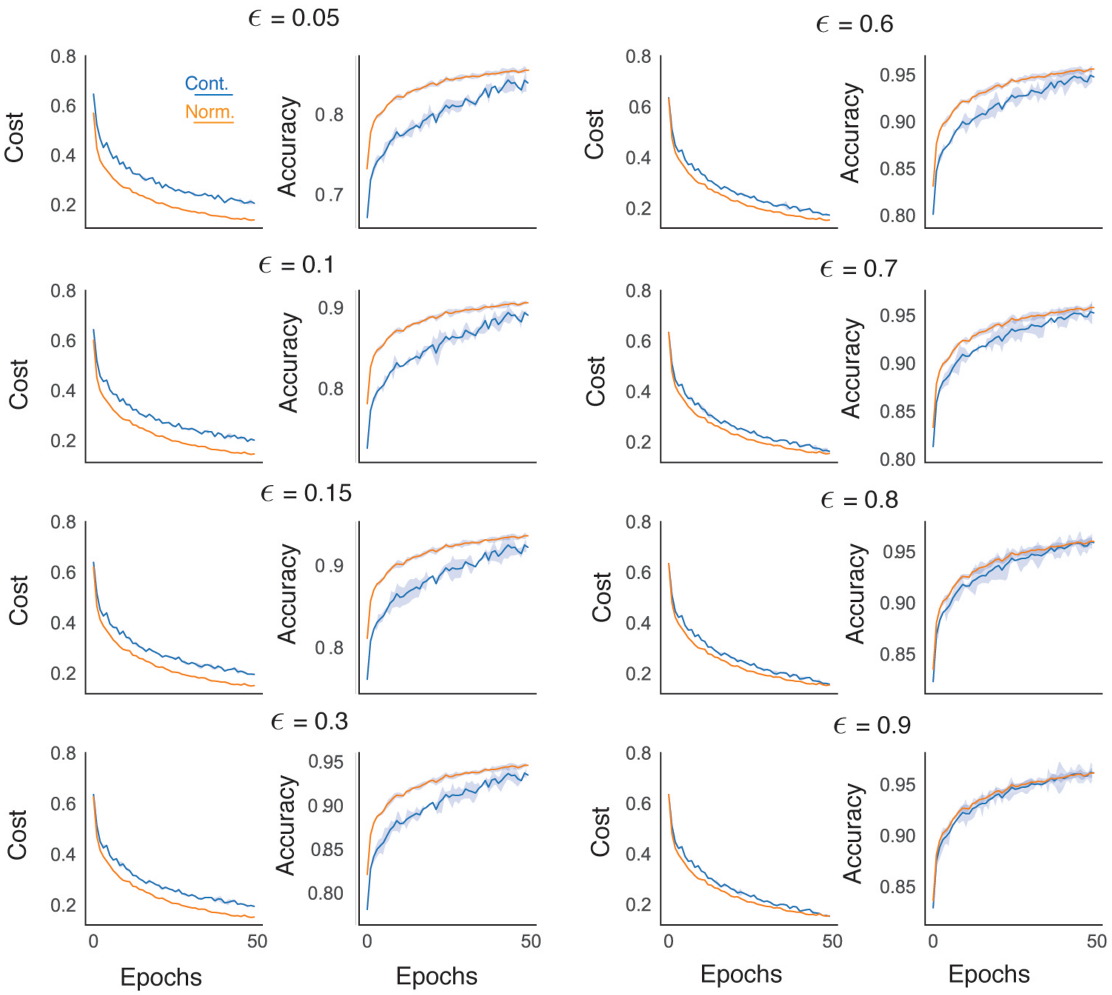

S1 Fig 1. Improved learning for different levels of sparsity. Training cost and test accuracy for a hidden layer of 100 units on the MNIST digits dataset for different levels of sparsity $\epsilon = 0.05, 0.1, 0.15, 0.3, 0.6, 0.7, 0.8$, and 0.9. The control network is in orange and the dendritically normalised network is in blue in all panels. Solid lines are means of 10 repetitions. Shaded areas show one standard deviation above and below the mean. In all cases the rewiring parameter $\zeta = 0.15$.
