# Supplementary material for: Dendritic normalisation improves learning in sparsely connected artificial neural networks
Source: PLoS Comput Biol. 2021 Aug 9;17(8):e1009202. doi: 10.1371/journal.pcbi.1009202 (PMC8407571; doi:10.1371/journal.pcbi.1009202)
Supplement: S1 Code — Code in Python and Matlab necessary to reproduce the figures. Code for Figs 1–4 is written in Python 3.6. The networks in Figs 1, 2 and 4 are coded using the standard Numpy package, and the networks in Fig 3 make use of Keras with a TensorFlow backend (keras.io). The application of dendritic normalisation in Keras with TensorFlow allows for immediate inclusion in Keras-based deep learning models. The normalisation requires a custom layer, constraint, and optimiser. Fig 5 uses code written in Matlab 2020b, using the freely available Trees Toolbox package [69]. (ZIP) [file pcbi.1009202.s002.zip › Supplementary_code_without_data/fig/Fig_5_d.pdf]

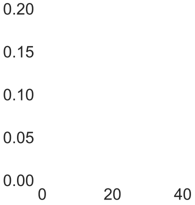
0.20
0.15
0.10
0.05
0.00
0
20
40
