# Supplementary figures and images for: Dendritic normalisation improves learning in sparsely connected artificial neural networks
Source: PLoS Comput Biol. 2021 Aug 9;17(8):e1009202. doi: 10.1371/journal.pcbi.1009202 (PMC8407571; doi:10.1371/journal.pcbi.1009202)

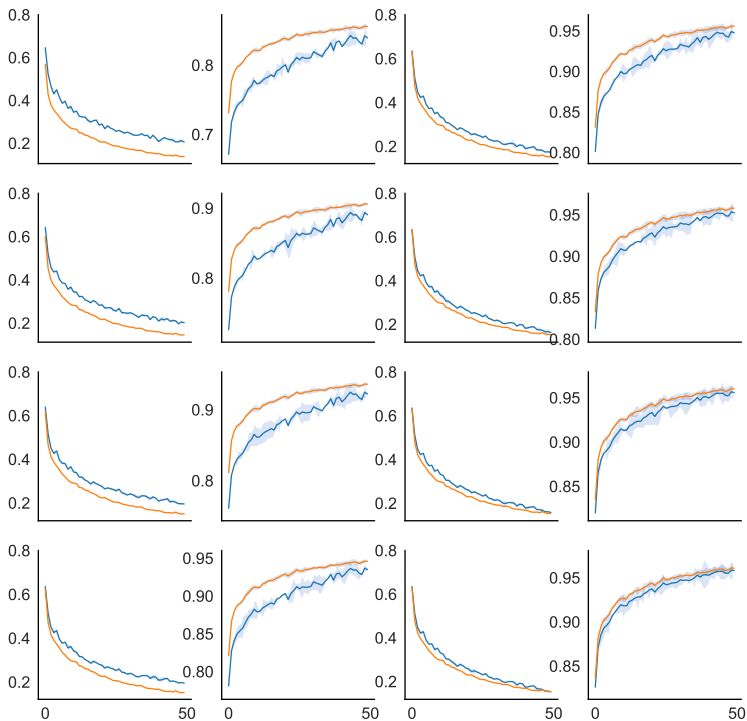

Supplement: S1 Code — Code in Python and Matlab necessary to reproduce the figures. Code for Figs 1–4 is written in Python 3.6. The networks in Figs 1, 2 and 4 are coded using the standard Numpy package, and the networks in Fig 3 make use of Keras with a TensorFlow backend (keras.io). The application of dendritic normalisation in Keras with TensorFlow allows for immediate inclusion in Keras-based deep learning models. The normalisation requires a custom layer, constraint, and optimiser. Fig 5 uses code written in Matlab 2020b, using the freely available Trees Toolbox package [69]. (ZIP) [file pcbi.1009202.s002.zip › Supplementary_code_without_data/fig/Fig_S1.pdf]

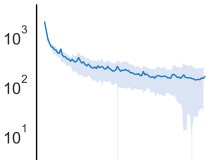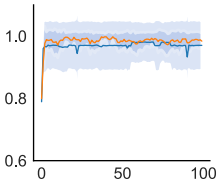

Supplement: S1 Code — Code in Python and Matlab necessary to reproduce the figures. Code for Figs 1–4 is written in Python 3.6. The networks in Figs 1, 2 and 4 are coded using the standard Numpy package, and the networks in Fig 3 make use of Keras with a TensorFlow backend (keras.io). The application of dendritic normalisation in Keras with TensorFlow allows for immediate inclusion in Keras-based deep learning models. The normalisation requires a custom layer, constraint, and optimiser. Fig 5 uses code written in Matlab 2020b, using the freely available Trees Toolbox package [69]. (ZIP) [file pcbi.1009202.s002.zip › Supplementary_code_without_data/fig/Fig_4_e_gd.pdf]

0.20

0.15

0.10

0.05

0.00

0.20

0.15

0.10

0.05

0.00

0

20

40

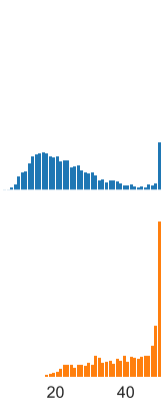

Supplement: S1 Code — Code in Python and Matlab necessary to reproduce the figures. Code for Figs 1–4 is written in Python 3.6. The networks in Figs 1, 2 and 4 are coded using the standard Numpy package, and the networks in Fig 3 make use of Keras with a TensorFlow backend (keras.io). The application of dendritic normalisation in Keras with TensorFlow allows for immediate inclusion in Keras-based deep learning models. The normalisation requires a custom layer, constraint, and optimiser. Fig 5 uses code written in Matlab 2020b, using the freely available Trees Toolbox package [69]. (ZIP) [file pcbi.1009202.s002.zip › Supplementary_code_without_data/fig/Fig_4_ci.pdf]

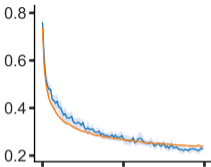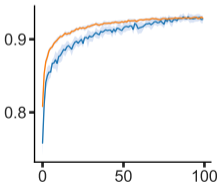

Supplement: S1 Code — Code in Python and Matlab necessary to reproduce the figures. Code for Figs 1–4 is written in Python 3.6. The networks in Figs 1, 2 and 4 are coded using the standard Numpy package, and the networks in Fig 3 make use of Keras with a TensorFlow backend (keras.io). The application of dendritic normalisation in Keras with TensorFlow allows for immediate inclusion in Keras-based deep learning models. The normalisation requires a custom layer, constraint, and optimiser. Fig 5 uses code written in Matlab 2020b, using the freely available Trees Toolbox package [69]. (ZIP) [file pcbi.1009202.s002.zip › Supplementary_code_without_data/fig/Fig_1_d_1.pdf]

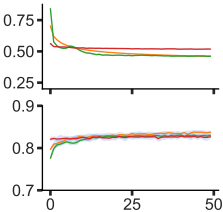

Supplement: S1 Code — Code in Python and Matlab necessary to reproduce the figures. Code for Figs 1–4 is written in Python 3.6. The networks in Figs 1, 2 and 4 are coded using the standard Numpy package, and the networks in Fig 3 make use of Keras with a TensorFlow backend (keras.io). The application of dendritic normalisation in Keras with TensorFlow allows for immediate inclusion in Keras-based deep learning models. The normalisation requires a custom layer, constraint, and optimiser. Fig 5 uses code written in Matlab 2020b, using the freely available Trees Toolbox package [69]. (ZIP) [file pcbi.1009202.s002.zip › Supplementary_code_without_data/fig/Fig_3_f.pdf]

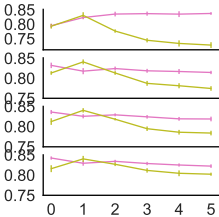

Supplement: S1 Code — Code in Python and Matlab necessary to reproduce the figures. Code for Figs 1–4 is written in Python 3.6. The networks in Figs 1, 2 and 4 are coded using the standard Numpy package, and the networks in Fig 3 make use of Keras with a TensorFlow backend (keras.io). The application of dendritic normalisation in Keras with TensorFlow allows for immediate inclusion in Keras-based deep learning models. The normalisation requires a custom layer, constraint, and optimiser. Fig 5 uses code written in Matlab 2020b, using the freely available Trees Toolbox package [69]. (ZIP) [file pcbi.1009202.s002.zip › Supplementary_code_without_data/fig/Fig_3_g.pdf]

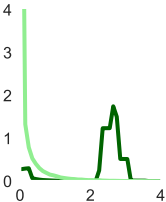

Supplement: S1 Code — Code in Python and Matlab necessary to reproduce the figures. Code for Figs 1–4 is written in Python 3.6. The networks in Figs 1, 2 and 4 are coded using the standard Numpy package, and the networks in Fig 3 make use of Keras with a TensorFlow backend (keras.io). The application of dendritic normalisation in Keras with TensorFlow allows for immediate inclusion in Keras-based deep learning models. The normalisation requires a custom layer, constraint, and optimiser. Fig 5 uses code written in Matlab 2020b, using the freely available Trees Toolbox package [69]. (ZIP) [file pcbi.1009202.s002.zip › Supplementary_code_without_data/fig/Fig_5_c.pdf]

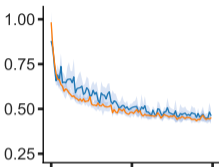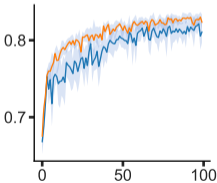

Supplement: S1 Code — Code in Python and Matlab necessary to reproduce the figures. Code for Figs 1–4 is written in Python 3.6. The networks in Figs 1, 2 and 4 are coded using the standard Numpy package, and the networks in Fig 3 make use of Keras with a TensorFlow backend (keras.io). The application of dendritic normalisation in Keras with TensorFlow allows for immediate inclusion in Keras-based deep learning models. The normalisation requires a custom layer, constraint, and optimiser. Fig 5 uses code written in Matlab 2020b, using the freely available Trees Toolbox package [69]. (ZIP) [file pcbi.1009202.s002.zip › Supplementary_code_without_data/fig/Fig_1_d_2.pdf]

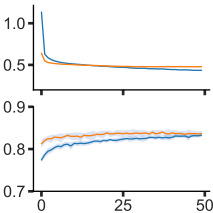

Supplement: S1 Code — Code in Python and Matlab necessary to reproduce the figures. Code for Figs 1–4 is written in Python 3.6. The networks in Figs 1, 2 and 4 are coded using the standard Numpy package, and the networks in Fig 3 make use of Keras with a TensorFlow backend (keras.io). The application of dendritic normalisation in Keras with TensorFlow allows for immediate inclusion in Keras-based deep learning models. The normalisation requires a custom layer, constraint, and optimiser. Fig 5 uses code written in Matlab 2020b, using the freely available Trees Toolbox package [69]. (ZIP) [file pcbi.1009202.s002.zip › Supplementary_code_without_data/fig/Fig_3_e.pdf]

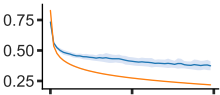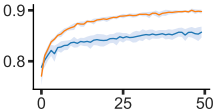

Supplement: S1 Code — Code in Python and Matlab necessary to reproduce the figures. Code for Figs 1–4 is written in Python 3.6. The networks in Figs 1, 2 and 4 are coded using the standard Numpy package, and the networks in Fig 3 make use of Keras with a TensorFlow backend (keras.io). The application of dendritic normalisation in Keras with TensorFlow allows for immediate inclusion in Keras-based deep learning models. The normalisation requires a custom layer, constraint, and optimiser. Fig 5 uses code written in Matlab 2020b, using the freely available Trees Toolbox package [69]. (ZIP) [file pcbi.1009202.s002.zip › Supplementary_code_without_data/fig/Fig_3_d.pdf]

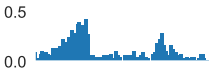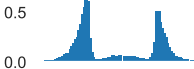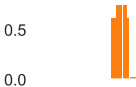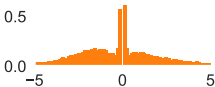

Supplement: S1 Code — Code in Python and Matlab necessary to reproduce the figures. Code for Figs 1–4 is written in Python 3.6. The networks in Figs 1, 2 and 4 are coded using the standard Numpy package, and the networks in Fig 3 make use of Keras with a TensorFlow backend (keras.io). The application of dendritic normalisation in Keras with TensorFlow allows for immediate inclusion in Keras-based deep learning models. The normalisation requires a custom layer, constraint, and optimiser. Fig 5 uses code written in Matlab 2020b, using the freely available Trees Toolbox package [69]. (ZIP) [file pcbi.1009202.s002.zip › Supplementary_code_without_data/fig/Fig_4_fii.pdf]

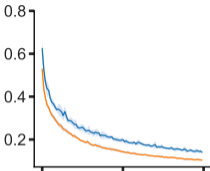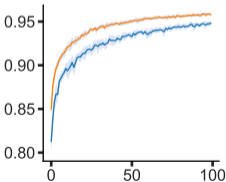

Supplement: S1 Code — Code in Python and Matlab necessary to reproduce the figures. Code for Figs 1–4 is written in Python 3.6. The networks in Figs 1, 2 and 4 are coded using the standard Numpy package, and the networks in Fig 3 make use of Keras with a TensorFlow backend (keras.io). The application of dendritic normalisation in Keras with TensorFlow allows for immediate inclusion in Keras-based deep learning models. The normalisation requires a custom layer, constraint, and optimiser. Fig 5 uses code written in Matlab 2020b, using the freely available Trees Toolbox package [69]. (ZIP) [file pcbi.1009202.s002.zip › Supplementary_code_without_data/fig/Fig_1_d_3.pdf]

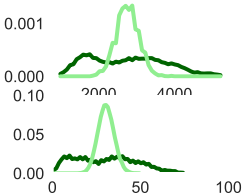

Supplement: S1 Code — Code in Python and Matlab necessary to reproduce the figures. Code for Figs 1–4 is written in Python 3.6. The networks in Figs 1, 2 and 4 are coded using the standard Numpy package, and the networks in Fig 3 make use of Keras with a TensorFlow backend (keras.io). The application of dendritic normalisation in Keras with TensorFlow allows for immediate inclusion in Keras-based deep learning models. The normalisation requires a custom layer, constraint, and optimiser. Fig 5 uses code written in Matlab 2020b, using the freely available Trees Toolbox package [69]. (ZIP) [file pcbi.1009202.s002.zip › Supplementary_code_without_data/fig/Fig_5_b.pdf]

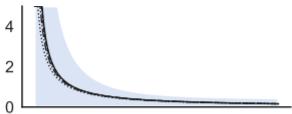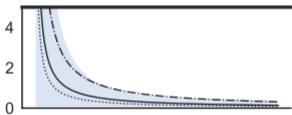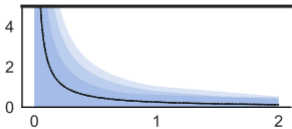

Supplement: S1 Code — Code in Python and Matlab necessary to reproduce the figures. Code for Figs 1–4 is written in Python 3.6. The networks in Figs 1, 2 and 4 are coded using the standard Numpy package, and the networks in Fig 3 make use of Keras with a TensorFlow backend (keras.io). The application of dendritic normalisation in Keras with TensorFlow allows for immediate inclusion in Keras-based deep learning models. The normalisation requires a custom layer, constraint, and optimiser. Fig 5 uses code written in Matlab 2020b, using the freely available Trees Toolbox package [69]. (ZIP) [file pcbi.1009202.s002.zip › Supplementary_code_without_data/fig/Fig_1_b.pdf]

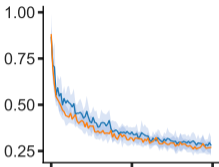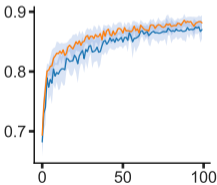

Supplement: S1 Code — Code in Python and Matlab necessary to reproduce the figures. Code for Figs 1–4 is written in Python 3.6. The networks in Figs 1, 2 and 4 are coded using the standard Numpy package, and the networks in Fig 3 make use of Keras with a TensorFlow backend (keras.io). The application of dendritic normalisation in Keras with TensorFlow allows for immediate inclusion in Keras-based deep learning models. The normalisation requires a custom layer, constraint, and optimiser. Fig 5 uses code written in Matlab 2020b, using the freely available Trees Toolbox package [69]. (ZIP) [file pcbi.1009202.s002.zip › Supplementary_code_without_data/fig/Fig_1_d_6.pdf]

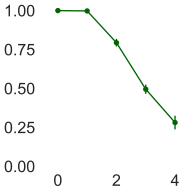

Supplement: S1 Code — Code in Python and Matlab necessary to reproduce the figures. Code for Figs 1–4 is written in Python 3.6. The networks in Figs 1, 2 and 4 are coded using the standard Numpy package, and the networks in Fig 3 make use of Keras with a TensorFlow backend (keras.io). The application of dendritic normalisation in Keras with TensorFlow allows for immediate inclusion in Keras-based deep learning models. The normalisation requires a custom layer, constraint, and optimiser. Fig 5 uses code written in Matlab 2020b, using the freely available Trees Toolbox package [69]. (ZIP) [file pcbi.1009202.s002.zip › Supplementary_code_without_data/fig/Fig_5_e.pdf]

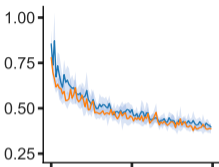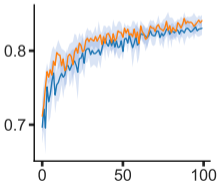

Supplement: S1 Code — Code in Python and Matlab necessary to reproduce the figures. Code for Figs 1–4 is written in Python 3.6. The networks in Figs 1, 2 and 4 are coded using the standard Numpy package, and the networks in Fig 3 make use of Keras with a TensorFlow backend (keras.io). The application of dendritic normalisation in Keras with TensorFlow allows for immediate inclusion in Keras-based deep learning models. The normalisation requires a custom layer, constraint, and optimiser. Fig 5 uses code written in Matlab 2020b, using the freely available Trees Toolbox package [69]. (ZIP) [file pcbi.1009202.s002.zip › Supplementary_code_without_data/fig/Fig_1_d_4.pdf]

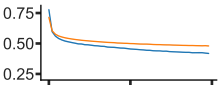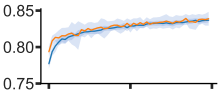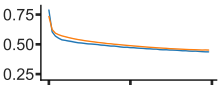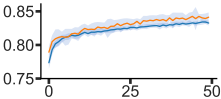

Supplement: S1 Code — Code in Python and Matlab necessary to reproduce the figures. Code for Figs 1–4 is written in Python 3.6. The networks in Figs 1, 2 and 4 are coded using the standard Numpy package, and the networks in Fig 3 make use of Keras with a TensorFlow backend (keras.io). The application of dendritic normalisation in Keras with TensorFlow allows for immediate inclusion in Keras-based deep learning models. The normalisation requires a custom layer, constraint, and optimiser. Fig 5 uses code written in Matlab 2020b, using the freely available Trees Toolbox package [69]. (ZIP) [file pcbi.1009202.s002.zip › Supplementary_code_without_data/fig/Fig_3_c.pdf]

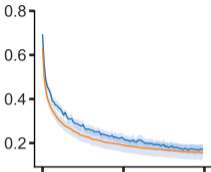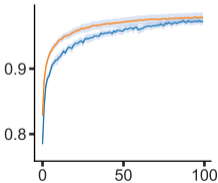

Supplement: S1 Code — Code in Python and Matlab necessary to reproduce the figures. Code for Figs 1–4 is written in Python 3.6. The networks in Figs 1, 2 and 4 are coded using the standard Numpy package, and the networks in Fig 3 make use of Keras with a TensorFlow backend (keras.io). The application of dendritic normalisation in Keras with TensorFlow allows for immediate inclusion in Keras-based deep learning models. The normalisation requires a custom layer, constraint, and optimiser. Fig 5 uses code written in Matlab 2020b, using the freely available Trees Toolbox package [69]. (ZIP) [file pcbi.1009202.s002.zip › Supplementary_code_without_data/fig/Fig_1_d_5.pdf]

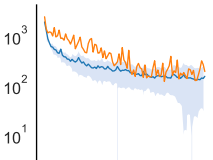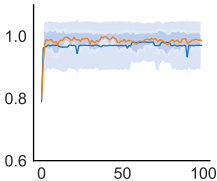

Supplement: S1 Code — Code in Python and Matlab necessary to reproduce the figures. Code for Figs 1–4 is written in Python 3.6. The networks in Figs 1, 2 and 4 are coded using the standard Numpy package, and the networks in Fig 3 make use of Keras with a TensorFlow backend (keras.io). The application of dendritic normalisation in Keras with TensorFlow allows for immediate inclusion in Keras-based deep learning models. The normalisation requires a custom layer, constraint, and optimiser. Fig 5 uses code written in Matlab 2020b, using the freely available Trees Toolbox package [69]. (ZIP) [file pcbi.1009202.s002.zip › Supplementary_code_without_data/fig/Fig_4_b.pdf]

0.20

0.15

0.10

0.05

0.00

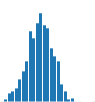

0.20

0.15

0.10

0.05

0.00

0

20

40

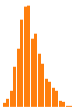

Supplement: S1 Code — Code in Python and Matlab necessary to reproduce the figures. Code for Figs 1–4 is written in Python 3.6. The networks in Figs 1, 2 and 4 are coded using the standard Numpy package, and the networks in Fig 3 make use of Keras with a TensorFlow backend (keras.io). The application of dendritic normalisation in Keras with TensorFlow allows for immediate inclusion in Keras-based deep learning models. The normalisation requires a custom layer, constraint, and optimiser. Fig 5 uses code written in Matlab 2020b, using the freely available Trees Toolbox package [69]. (ZIP) [file pcbi.1009202.s002.zip › Supplementary_code_without_data/fig/Fig_4_fi.pdf]

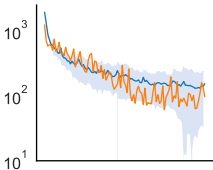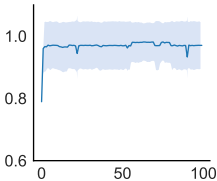

Supplement: S1 Code — Code in Python and Matlab necessary to reproduce the figures. Code for Figs 1–4 is written in Python 3.6. The networks in Figs 1, 2 and 4 are coded using the standard Numpy package, and the networks in Fig 3 make use of Keras with a TensorFlow backend (keras.io). The application of dendritic normalisation in Keras with TensorFlow allows for immediate inclusion in Keras-based deep learning models. The normalisation requires a custom layer, constraint, and optimiser. Fig 5 uses code written in Matlab 2020b, using the freely available Trees Toolbox package [69]. (ZIP) [file pcbi.1009202.s002.zip › Supplementary_code_without_data/fig/Fig_4_e.pdf]

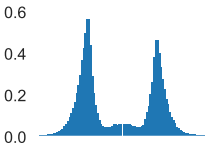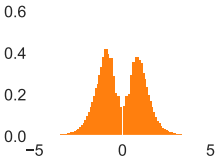

Supplement: S1 Code — Code in Python and Matlab necessary to reproduce the figures. Code for Figs 1–4 is written in Python 3.6. The networks in Figs 1, 2 and 4 are coded using the standard Numpy package, and the networks in Fig 3 make use of Keras with a TensorFlow backend (keras.io). The application of dendritic normalisation in Keras with TensorFlow allows for immediate inclusion in Keras-based deep learning models. The normalisation requires a custom layer, constraint, and optimiser. Fig 5 uses code written in Matlab 2020b, using the freely available Trees Toolbox package [69]. (ZIP) [file pcbi.1009202.s002.zip › Supplementary_code_without_data/fig/Fig_4_cii.pdf]
