# Supplementary material for: Dendritic normalisation improves learning in sparsely connected artificial neural networks
Source: PLoS Comput Biol. 2021 Aug 9;17(8):e1009202. doi: 10.1371/journal.pcbi.1009202 (PMC8407571; doi:10.1371/journal.pcbi.1009202)
Supplement: Attachment — Submitted filename: Response_to_review.pdf [file pcbi.1009202.s003.pdf]

Response to review

Reviewer #1:

*In the Introduction it is unclear when findings relate to feedforward vs recurrent networks. For example, small-worldness (as I know it) is a property of recurrent networks.

We thank the reviewer for this comment. We now specify in the Introduction that these properties are observed in recurrent networks. We have further substantially expanded our Results, showing the application of dendritic normalisation to both recurrent networks trained with backprop (Figure 4) and spike-timing dependent plasticity (Figure 5). This further clarifies the effects of SET and dendritic normalisation within a recurrent network.

*The SET algorithm needs to be explained better, and more so in the main text. The methods section says "After each training epoch, a fraction of the weakest contacts are excised and an equal number of new random connections are formed". Is this specific to each neuron such that the number of connections per neuron doesn't change or is it that the number of connections in the entire network is conserved? If it is the former then the normalization would simply be division by a (neuron-specific) constant, which I thought may be the case given the statement on line 94. But then Figure 2B shows contacts per neuron changing over training so I assume it is the latter. This is an important point to be clear on.

We have brought our explanation of SET back into the Results and clarified that the total number of connections in the network remains constant, but that the number of connections received by each neuron may vary.

*I am a bit confused as to how the different normalizations compare and what the authors are claiming about their dendritic normalization.

For example: "The normalisation appears to make better use of neural resources by distributing connections and weights more evenly across the available cells, whilst keeping expected inputs closer to 0, the steepest part of the activation function, where responses are most sensitive to inputs"
First, does "whilst keeping expected inputs closer to 0" align with what's plotted? The distribution for the normalized model in the right column of Figure 2B starts centered around 0 but spreads out with training such that is seems like most cells get input far from 0, similar to the unnormalized model. Also, is this the main explanation of the benefit of the L0 norm? How does this compare to the benefit of the L2 norm? That is, are the authors claiming that the L0 norm is having a qualitatively different effect than L2 that makes the L0 norm beneficial in its own way?

We agree that in the final epochs, most inputs are relatively far from zero in both cases, although slightly closer in the normalised case. We clarify this and have expanded our later explanation of the comparison with the L2 norm.

"In consequence, cells receiving a few strong connections will be relatively fast-learning and unstable compared to those that receive many, individually less effective, connections." Doesn't the L2 norm mean that cells with strong connections will learn slowly? In fact, it seems like the L0 and L2 norms fight each other (especially if overall weight is conserved). It may be helpful to simply plot a measure of the gradients for the weights a cell receives as a function of the number of contacts a cell gets and/or its overall weight input, for the different norm types. That way the differences between L2, L0, and the combination can be clearly and empirically shown.

We agree that here both types of connectivity, with relatively few strong and many weak contacts, will potentially produce similar learning speeds. For comparison with the L2 norm, we have added a panel (Fig 3f) showing the relative gradient magnitudes as a function of both the number and weight of connections. This allows us to better illustrate the differences between the norms and we thank the reviewer for this suggestion.

"As neurons with many weak connections learn relatively slowly when both normalisations are in place it appears that good use is made of existing connectivity, but that less informative connections are not selectively weakened enough to be excised by the SET algorithm." I find this statement a bit hard to parse. Is it just saying that the slow learning essentially means that the SET part of the learning process isn't relevant? Also, why wouldn't this be true for L0 alone?

This wouldn't necessarily be true for the L0 norm alone as the learning rate would be independent of the strength of the individual connections. We have clarified this point, referencing the new panel Fig 3f.

"The joint mechanism appears well suited to sparse, but static, connectivity" It seems like the joint mechanism results in static connectivity (and worse performance). I don't know if that makes it well suited for that, per se.

Over the first epoch, the connectivity is static and here the joint norm has lower cost and higher accuracy than either individual norm. We have clarified this point.

*" The dendritic normalisation (L0-norm in pink) has the highest performance after 10 epochs, although the L1-norm with variable excitability is similar. " Why do the authors report on 10 epochs when training goes to 20? Is this statement true for 20 as well? Can you include in-line

here the performance +/- SD and whether this is significantly different from the next best one. It's not so easy to see on the graph.

We have clarified that the situation is similar after 20 epochs and have included standard deviations and a significance test.

*"here the two streams of input would be in competition as more strongly recurrently connected cells would receive relatively weak feedforward inputs and vice versa." why would this be?

This would occur because each additional contact would reduce the weight of the other existing contacts. The new results for recurrent networks around Figure 4 deal with this issue explicitly.

*Overall, I am struggling to find a clear demonstration of what is unique and relevant about the L0 norm. It seems to lead to relatively similar performance as L2 and aside from some brief suggestions about what the differences may be, there isn't any actual analysis of how L0-normed networks learn differently than L2 ones (while it is interesting that the L0 norm has its own biological motivation, that is not enough.). If the L0 norm is just doing something that is mostly the same as any other norm (i.e., keeping weights within a reasonable range) then I'm not sure how interesting it is to the biological community. The authors even say that "The comparison between the heterosynaptic plasticity-like L2-normalisation and our dendritic L0-normalisation is particularly interesting." I agree, but I don't feel that this is actually done to a great extent in the paper, aside from some speculating. Given that this is a computational biology journal rather than a machine learning one, I think the authors need to put in more effort to provide some insights into how the system works, and also how this relates to how real neurons work. This is especially important if the main benefit of the normalization is how it controls learning rates, given that the learning algorithm (backprop) is not directly relatable to biological learning. That is, the authors should be able to clearly state what they believe the implications from this somewhat non-biological learning are for biological learning.

We have substantially expanded the results of the paper to explicitly illustrate the biological relevance of the L0 norm. This includes a section laying out the justification in more detail (leading to panel 1b) and another illustrating the normalisation occurring naturally in spatially-extended model neurons (Figure 5). We have further expanded Figure 3 to better illustrate the comparison of different norm types and more improved our discussion of these results in line with the individual comments above. We feel that the paper in its revised format better illustrates the importance of the L0-norm for biological neurons. We agree that the mechanism of improved learning is not hugely dissimilar from that provided by other norms in keeping activations within a critical range, but argue that dendritic normalisation is interesting due to both the fact that it arises naturally in dendrites (and neurons would have to expend additional energy to counteract it) and that it does appear to be particularly

suited to sparse networks. In addition, to make the link between neurobiology and machine learning algorithms more clear, we have added new references from neuroscience about experimental evidence for heterosynaptic plasticity and synaptic normalisation.

I should also point out that one of the main contributions ("a practical normalisation procedure that drastically improves learning in sparse artificial neural networks trained using backpropagation.") seems to not hold when compared to existing L2 norms.

We have replaced 'drastically' with 'significantly' and justified this with the T-test mentioned above. We would also note that many of the papers studying sparse networks do not currently use any normalisation.

Minor Comments:
It's difficult to see the shading in Figure 1d

We have made the shading darker.

Line 163: what is this sigma? I don't see it in the equations or previously referenced.

Sigma should have been 'g'; we have now corrected this.

Reviewer #2:

The authors investigate the benefits of normalizing synaptic weight strength by the number of postsynaptic connections in sparse neural networks with evolving connectivity. More concretely, the authors consider standard artificial feedforward neural network models trained with end-to-end stochastic gradient descent; an additional connectivity remodeling process ("SET") replaces weak connections by random new ones as training proceeds. The proposed normalization is a natural extension of weight normalization techniques to the sparse, non-homogeneous connectivity case. As the authors comment in the abstract, their research essentially results in "improved machine learning tools".

While I appreciate the simplicity of the method from a practical machine learning perspective, I feel that the paper is not very well matched to the PLOS Computational Biology readership. There is an interesting connection to previous biophysical modeling work from the same group, but I think it is fair to say that the normalization can be easily derived from purely theoretical considerations. I find it hard to call this work "bio-inspired machine learning". Apart from this connection, the research is essentially standard machine learning research.

Thus, I would recommend submitting this work to an artificial neural networks venue with less emphasis on biology than PLOS Computational Biology, or thoroughly rewriting the

paper having a non-machine-learning readership in mind. Admittedly, it may be hard to do so.

We thank the reviewer for this comment. We have incorporated new results that better show the biological motivation for the dendritic normalisation. In particular, we have added a subsection to the beginning of the Results entitled 'Dendritic normalisation is an intrinsic property of spatially extended neurons' where we derive the normalisation in terms of cable theory and plot its effect for different soma sizes and synaptic time constants in the new panel b in Figure 1. We further apply the normalisation to recurrent networks trained with backpropagation and show the effects on mean shortest path lengths in the recurrent layer (the new Figure 4). Finally, we consider a network of spatially extended neurons equipped with a spiking mechanism and undergoing self-organisation through spike-timing dependent plasticity; in this case the presence of dendrites allows the network to reach a stable state that is not possible otherwise (the new Figure 5).

We agree that many of our major results are comparable to those in a standard machine learning framework, but feel that this is a striking way to illustrate the computational impact of dendritic normalisation. We have rewritten the Abstract entirely and sought throughout the paper to better link our results to biological phenomena. We hope that this better highlights the biological implications of our work whilst maintaining the links to existing machine learning techniques.

I leave some additional comments to the authors below:

- I suppose that the authors are aware that as a machine learning paper, the experiments are lacking by today's standards in the choice of architectures and range of problems considered. It may be worth considering recurrent neural networks, given the difficulties of optimizing them and their relevance in computational neuroscience.

We agree that our results are not comparable to those of today's densely connected deep networks, but argue (and demonstrate in Table 1) that they are equivalent or superior to those published for sparse feedforward networks. We have also expanded our results to consider recurrency, both trained with backpropagation (Figure 4) and self-organised (Figure 5).

- The most surprising result, for me, was that constant excitability was beneficial (around l. 200). The paper would be substantially stronger if the authors provided more experimental evidence in favor of this finding, as the per-neuron excitability is usually featured in batch and weight normalization and believed to be beneficial to improve optimization in deeper and more complex neural network models.

We typically find constant excitability to be superior to per-neuron excitability (Figure 3h). We have expanded our description of this phenomenon in the Results; we believe that constant excitability is beneficial as it both regularises the system, allowing better generalisation to unseen data, and incorporates the entirety of the error signal into refining the excitability and therefore effective gradients (Eq 6). We have highlighted this as an interesting future direction of study, but believe a

detailed exploration would be tangental to this paper given that there are a large number of intermediate cases (subpopulations of different sizes, differences between layers) to consider.

- It would be good to know how the interplay between the spherical and L0 weight normalizations is affected when considering less sparse, but still SET-rewired networks. A related question, how do the hyperparameters of the SET algorithm, which were kept fixed here, affect the results?

We have added a supplementary figure showing a scan over different sparsities. The results are stronger for sparser and weaker for less sparse networks.

- Why are the contact distributions bell-shaped (Fig. 2) and far from the more interesting "small-world and scale-free topologies similar to biological neuronal circuits (31)" (l. 59) referred to by the authors?

The distributions are bell-shaped in this case as they are for purely-feedforward connections. We have clarified in the Introduction that small-world and scale-free distributions arise in recurrent networks. Our results in Figure 2 in the control case are in line with those previously published with the SET algorithm for feedforward networks. We have additionally added new results on recurrent networks with dendritic normalisation, where path lengths are typically reduced from the initial case (Figures 4 and 5).

- The authors conclude the abstract by claiming that their method "renders [sparse networks] better understood and more stable in our tests", after stating that artificial neural networks "result in poorly understood parameters". After reading the paper I was puzzled by these claims, as I couldn't see why the proposed normalization is related to any improved parameter interpretability.

We have rewritten the abstract entirely to better explain the manuscript.

Reviewer #3: Summary:

Through simulations of artificial neural networks, this work makes explicit that having dendrites constraining synapses to scale their weight with the inverse of their number (i.e. normalization) comes with foreseeable advantages for learning, when learning synapses learn according to the backpropagation-of-error algorithm. The advantages are mainly in allowing for a faster progression of learning and are robust to the type of normalization, the number of neurons in a layer and the depth of the network.

Assessment:

I found myself oscillating between the excitement at seeing a clear function of all dendrites for learning and the deception of the fact that the paper is a small adaptation recast with an introduction on dendrites that weight normalization may be beneficial for learning. I think that a revised presentation can mitigate the deception.

Some important criticisms:
1. Rationale. The paper is evasive about the rationale and the result section has none. From what I can see, the simulations are exactly as in some other ML papers, with the exception of combining normalisation and sparsity. The reader appreciates that these papers are presented form the get-go, but we are missing a rationale for delving into the variants studied here. Why do we need to focus on sparse nets? Do we expect anything different or is this a simple sanity check for this more biologically realistic constraint? If it is studied for biological realism, why any of the other issues with biological realism, such as those mentioned in the discussion. There is more rationale given in the discussion (li 275, etc) than in the results section.

The necessity of studying this normalisation in sparse networks comes from the fact that the L0 norm will only vary if neurons have different numbers of afferent connections. In a fully-connected network, the normalisation presented here would have no effect. We have added a sentence in the Introduction to clarify this.

2. Gradient-based learning. The fact that these results are tied to training with backprop should be ideally part of the rationale. The results presented here would hold only if the neurons are actually learning with backprop. There is work in this direction (see Sacramento et al. 2018, Payeur et al. 2020, Bellec et al. 2020; and these papers are clearly making the claim that it is backprop can be realistic, so it is (thankfully for the present paper) not true that the gradient information is necessarily unavailable to synapses as mentioned in the discussion and introduction).

We chose to focus on backpropagation as it is the most effective way to tune connections given a learning task and uses all available information about the cost gradient. We found that the normalisation presented here is particularly general and so wanted to highlight that it allows better learning in an ideal case. The results should also attract wider interest. We have expanded the Results to show an example of the normalisation working alongside spike-timing dependent plasticity and also discuss the biological implementation of backpropagation in the Discussion. In addition, in line with the point of the reviewer, we have added new references showing that neurons might use backprop.

3. Validity of the premises. The entire paper being based on the idea that dendrites implement a normalization. Now for this fact, the authors reference their prior work on bioRxiv and the reader is supposed to just take this for granted. But having read the reference, I find it less than obvious that this is necessarily the case, or that the referenced paper actually serves a good reference for this fact since it is not the main point of that paper. The reference does not have an explicit figure on normalisation, something along the line of effective weight against number of synapses. Or if there is one, I did not get it. We therefore do not know the level with which this is an accurate picture of the biophysical properties of dendrites, we do not know where this approximation breaks down nor if it does at all. Further, a more solid establishment of this paper in the biological--as opposed to the machine learning--realm is

specifically what is lacking from the narrative. In thinking about this, I am not able to see why this normalization property has be down to dendrites only. So there is an inference step that the authors are making in establishing the premises of the study that is not obvious. I find the absence of a specific validation of the premises concerning and I am sure that other readers will have the same worry. (I would even go so far as to say that the claim in the introduction that ‘we show how the dendritic morphology of a neuron produces an afferent weight normalisation’ is blatantly wrong).

We agree that the link to our prior work was not made explicitly enough as that paper focussed on overall excitability and not the contribution of individual synapses. As this point is so crucial to our present manuscript we have added a subsection to the beginning of our results that presents a derivation of the normalisation in terms of cable theory (Eqs 1-3 & 10-18) and shows that it arises from the passive properties of a dendrite in a number of cases (Fig 1b).

We further agree that such a normalisation would not have to down to dendrites alone and could be implemented by any number of neuronal mechanisms; our point is instead that dendrites alone naturally produce an effective L0 normalisation. We have expanded the section in the Discussion where to compare passive normalisation to heterosynaptic plasticity to make this clearer.

Minor points

1. I did not understand the content of the paper from the abstract. For instance, there is no mention of dendrites. Learning comes out of the blue. The paper is said to be about introducing a normalisation but that would be missing the point.

We have rewritten the abstract to make our points more clearly.

2. The introduction does not situate this work as being part of a number of other studies in finding a specific network-level function of dendrites. Instead, the introduction focused more on the details that make ANNs slightly better.

We feel that the focus of the Introduction is appropriate as it lays out the literature on effective ANNs alongside the biology of dendrites and this is most necessary to provide context for the novel results in the paper. We believe that dendrites have a large number of computational roles in a network, which we cover in the Discussion. We will be happy to move this material to the Introduction if it is deemed necessary, but we hope that the rewritten abstract, alongside the expanded Results, will best situate our work.

4. Ambiguous statement on the nature of the normalisation. The abstract implies that it is normalised by ‘the number of active inputs’ and Eq. 1 states something similar with the L0 norm (but I am confused as vi is not defined, is it activity or some type of raw weight?). But then much of the text is about the sheer number of connections.

We agree that this statement in the Abstract is ambiguous as it is only applies when individual weights are of a similar size. We have rewritten the entire abstract to make our point clearer and have defined vi below (the old) Eq 1.

5. The vector vi is not defined.

We have defined this below the equation.

6. The cost function C is not defined.

We now specify that this holds for any differentiable cost function and that we define the ones used for the different figures in the Methods..

7. Eq. 2 seems wrong. Perhaps an explanatory step is needed: the gradient over v should give two terms when applied to equation 1 because v_i appears in both in itself and in the normalisation.

The equation takes this form as the L0 norm of v_i is constant almost everywhere with respect to v_i. We now mention this to avoid confusion.

8. Li 105 states that the networks are trained with SET, but that is not entirely true: missing that they are trained with SET and SGD with backprop.

We have clarified this.

9. The 'performance' or the statements that 'learning is improved' is not clearly defined and seem to oscillate between meaning accuracy and learning speed. Please make more precise throughout.

We have clarified our meaning in each case.

10. The comparison on accuracies is not very convincing between normalized and unnormalized as many networks have not finished learning, muddling learning rate and accuracy together.

We have clarified that these results typically focus on learning performance.

11. The point about the greater reliability is not illustrated clearly, the difference in error bars is nor consistent nor obvious (it seemed to be violated in Fig. 3).

This is also reflected in the joint feedforward-recurrent task in the new Figure 4. We have removed this claim.

12. More explanation as to why we expect L2 norm to be implemented by heterosynaptic mechanisms is needed. As it stands, it is as though these two processes are synonymous.

We agree that we were careless with our language after introducing this analogy. We now explicitly distinguish between the biological process and the artificial normalisation.

13. Li 146 'the improvement seems to increase with complexity'. This vague statement should either be established firmly (I mean the simulations are there) or removed.

We agree that this statement is vague, it was intended to apply to both convolutional and deeper architectures with sparse layers. We have removed it.

14. Li 199 ‘All normalisation show substantial improvement over the control case (Fig 1d)’ I did not understand what Fig 1d had to do with this, probably meant 3g, but then that is not what 3g shows either.

The reference to Figure 1 is correct, as this shows a comparable unnormalised network performing the same learning task. We have now clarified this.

15. Fig. 3g seems out of place in figure 3 since it is about something entirely different than depth (the title of figure 3) and the idea of looking at different types of normalization would need to be fleshed out.

In response to this and comments from other reviewers, we have expanded Figure 3 with an additional panel that compares the gradient magnitudes arising from different norms in terms of the number of afferent connections and their mean weight. We feel that this is a better introduction to the comparison of norm orders in the final two panels. We have changed the title of the figure to reflect this.

16. Li 290 ‘drastically improves’ is an overstatement.

We have changed ‘drastically’ to ‘significantly’ and justified this by assessing statistical significance of the improvement with a Welch’s T-test in the Results..
