# Supplementary material for: Dendritic normalisation improves learning in sparsely connected artificial neural networks
Source: PLoS Comput Biol. 2021 Aug 9;17(8):e1009202. doi: 10.1371/journal.pcbi.1009202 (PMC8407571; doi:10.1371/journal.pcbi.1009202)
Supplement: Attachment — Submitted filename: Response_to_review.pdf [file pcbi.1009202.s004.pdf]

Response to review

Reviewer #1:
I appreciated the added effort the authors have put into the work, particularly the recurrent architectures and spiking network. However there are still some areas that need corrections/clarification.

We would like to thank the reviewer for their time and comments. We believe that we have been able to correct all remaining points (see below).

This statement is added to the Introduction and it is not clear to me what part of the results it refers to. Is it just trying to say that the experiments use backprop? It should be made more clear: "secondly we demonstrate that learning is improved by dendrites in the ideal case where all gradient information is available to every synapse."

This statement was intended to provide additional rationale for using backprop when the normalisation mechanism is biologically derived. We have clarified this, adding 'as is the case with the traditional backpropagation algorithm'.

This statement (now in the Results section) has still not been clarified to say that this is happening over the whole network, rather than individual neurons:
"After each training epoch, a fraction ζ of the weakest contacts are excised and an equal number of new random connections are formed. New connection weights are distributed normally with mean 0 and standard deviation 1."

We have altered this statement to read 'After each training epoch, a fraction ζ of the weakest contacts in the entire layer are excised and an equal number of new random connections are formed between different neurons. This means that the number of connections received by each neuron will typically change between epochs.'

Furthermore, if the number of connections per neuron is changing why do the authors say it is almost everywhere constant in line 132?

We intended to say that the norm is almost everywhere constant as it is only changed by the evolutionary step, not through gradient descent. We have amended this statement to read 'Note that |v_i|_0 is almost everywhere constant during gradient descent as it is the number of non-zero elements of v_i (although this may change between epochs under the evolutionary connectivity algorithm)'.

The authors highlight when discussing the results of Figure 1 that the benefits to learning

from normalization can be seen not just in test accuracy, but also in training cost and in variability over random initializations. Only one of these (benefits as measured on test accuracy) holds in Figure 3, yet the authors say "In all cases, learning is improved by dendritic normalisation (orange versus blue lines in Figures 3b and d for the MNIST-Fashion dataset) in a similar manner to that for the single-layered networks described above." This is simply not true and needs to be rewritten in a way that accurately describes the figure.

We have altered this statement to read 'In all cases, final classification performance and training speed are improved by dendritic normalisation (orange versus blue lines in Figures 3b and d for the MNIST-Fashion dataset). Interestingly, the reduction in trial-to-trial variability seen in single-layer networks (Figure 1) does not occur here.' We feel this is a more accurate reflection of the results in figure 3.

The claim on lines 390-393 needs to be made more explicit, i.e. say what the performance of that algorithm is compared to this one.

This claim was intended to refer to the different normalisations allowing the network to converge, unlike in the unnormalised case. SORN in the original paper is implemented with both L1 normalisation and homeostatic changes in the threshold to maintain firing rates and it is not clear how far each feature contributes to their results. We have clarified our original statement to 'achieves a qualitatively similar result in allowing the network to converge to a stable state'.

line 319: "network network" typo

We have corrected this.

Reviewer #3:

"Dendritic normalisation improves learning in sparsely connected artificial neural networks" was quite seriously and adequately revised. The authors addressed all of my concerns. I find the article reads much better and its interest for the computational neuroscience is clear and convincing. The added derivation at the beginning is excellent and fits very well. The addition of BPTT makes it quite impressive. I commend the authors for the careful and exhaustive revision.

We would like to thank the reviewer for their time and comments. We believe that we have been able to correct all remaining points (see below).

Two minor comments:
In the (new) first 2 paragraphs of the results section, it should be stated early on that the

derivation is for the stationary state. Along those lines, it can be nice for the reader that the mean and variance (Eq 2) are over spatial configurations of the synapses.

We have modified the description of the first derivation to read 'The steady-state voltage at the root in response to a constant current influx' and added '(over all possible synaptic locations)' to describe the moments in Eq 2.

The performance of 90% (reported in the figures) for MNIST is on the low side for a 3 layer network. But then the table speaks of an accuracy of 99% in the table. It would be nice to have a clear explanation of the mismatch between the accuracy in the table and in the figure.

The networks with 90% accuracy are not especially large given their sparse connectivity and have relatively few parameters. The performance in Table 1 comes from copying the larger architectures employed by the cited papers for a fair comparison. We have rewritten the caption as 'Table of performance for benchmark datasets compared to published results on sparse networks. We replicate the published architecture in each case for a fair comparison...' and added 'in each case replicating the published network size and hyperparameters' to the results to clarify this.

I still think that the field as changed such that the phrase 'local algorithms are still regarded as a more plausible model for training networks' does not holds, particularly when used in opposition to some of the biological approximation to backprop. All of the biological approximations to backprop are meant to give a local algorithm. Perhaps the authors meant something closer to the ML 'unsupervised'?

We agree that this statement was misleading and have rewritten it as 'a variety of other learning algorithms are also regarded as biologically plausible models for training networks' before moving on to the discussion of spike timing in the next sentence.
